# Incidence of soft tissue releases in robotic assisted cementless TKA with mechanical alignment and flexion gap balancing

**DOI:** 10.1186/s42836-023-00188-1

**Published:** 2023-06-07

**Authors:** Nanchappan Selvanathan, Femi E. Ayeni, Rami Sorial

**Affiliations:** 1 Department of Orthopaedics, Nepean Hospital, Derby Street, Kingswood, Penrith, NSW 2747 Australia; 2 Nepean Institute of Academic Surgery, Nepean Clinical School, The University of Sydney, 62 Derby St, Kingswood, NSW 2747 Australia

**Keywords:** Robotic-assisted total knee arthroplasty, TKA, ROSA, Mechanical alignment, Soft tissue release

## Abstract

**Background:**

To ensure the success of total knee arthroplasty (TKA), precise bone cuts and a well-balanced soft tissue envelope are crucial. Soft tissue release may be necessary, subject to various factors. Therefore, documenting the type, frequency, and necessity of soft tissue releases can establish a benchmark for comparing different alignment techniques and philosophies and evaluating their outcomes. The purpose of this study was to demonstrate that robotic-assisted knee surgery requires minimal soft tissue release.

**Methods:**

We prospectively documented and retrospectively reviewed the soft tissue releases employed in securing ligament balance in the first 175 patients who received robotic-assisted TKAs at Nepean Hospital. ROSA was utilized in all surgeries with the aim of restoring mechanical coronal alignment, with a flexion gap balancing technique. Surgeries were performed between December 2019 to August 2021 by a single surgeon who used a standard medial parapatellar approach without a tourniquet, and the cementless persona prosthesis. All patients were followed up for a minimum of 6 months post-surgery. Soft tissue releases included any form of medial release for varus knee, posterolateral release for valgus knee and PCL fenestration or sacrifice.

**Results:**

There were 131 female and 44 male patients, aged between 48 to 89 years (average 60 years). The preoperative HKA ranged from 22 degrees varus to 28 degrees valgus, with 71% of patients presenting with a varus deformity. For the whole group, the no need for soft tissue release was documented in 123 patients (70.3%), small fenestrated releases of PCL in 27 (15.4%), sacrifice of PCL in 8 (4.5%), medial releases in 4 (2.3%) and posterolateral releases in 13 (7.4%). In 29.7% of patients in whom a soft tissue release was necessary for balance, over half were/received minor fenestrations of the PCL. Outcomes to date included no revisions or impending revisions, 2 MUAs (1%), and Oxford knee scores averaged 40 at 6 months.

**Conclusion:**

We concluded that Robot technology enhanced the precision of bone cuts and allowed for titration of required soft tissue releases to achieve optimal balance.

## Introduction

Robot-assist TKA allows for execution and validation of higher precision of bone cuts [1]. It also makes it possible to intraoperatively assess soft tissue balance objectively, which is critical to a ‘balanced and stable knee’. It is important to achieve a balanced knee both clinically and in terms of robotic parameters.

Information obtained enables surgeons to gauge the outcome of knee balancing before and after bone resection. Digital information prevents/avoids redundant bone cuts and soft tissue release. Moreover, from the ‘surgeon’s feel’ of a balanced knee, which has been demonstrated to be less than accurate, current technologies can allow for digital representation of soft tissue laxity and inform the required soft tissue releases to achieve balance. The combination of clinical assessment and digital parameters enables surgeons to evaluate and respond to the required actions needed to attain better balance.

Managing a severe coronal plane knee deformity using conventional instrumentation may require significant soft tissue release, which may be compounded by relative inaccuracy of the tibial or femoral bone cuts, thereby carrying a risk of over-release and instability [2]. Documenting and analyzing the number and extent of soft tissue release with each technology and alignment philosophy may assist in working towards the most appropriate and reproducible technique to achieve balance with the least invasiveness and thereby improve patient outcomes further [3]. Thus, this study sought to investigate if minimal to no soft tissue release is required in robotic-assisted knees.

## Methods

We prospectively documented and retrospectively reviewed the soft tissue release required in securing ligament balance in the first 175 consecutive robot-assisted TKAs in our centre. The robotic surgical assistant ROSA (Zimmer Biomet, Warsaw, IN, USA) was used in all surgeries, aiming for restoring mechanical alignment with HKA of 0 and a flexion gap balance by using the ligament tensioning “FuZion” device (Zimmer Biomet, Warsaw, IN, USA). Surgeries were performed between December 2019 to August 2021 by a single surgeon using a standard medial parapatellar approach without a tourniquet. All implants were cementless Persona TKA prosthesis (Zimmer Biomet, Warsaw, IN, USA) and all patients were followed up for a minimum of 6 months post-surgery. Soft tissue releases took into account any form of medial side release for varus knee, posterolateral fenestrated release and/or lateral retinacular release for valgus knee and PCL fenestration or sacrifice.

**Fig. 1:**
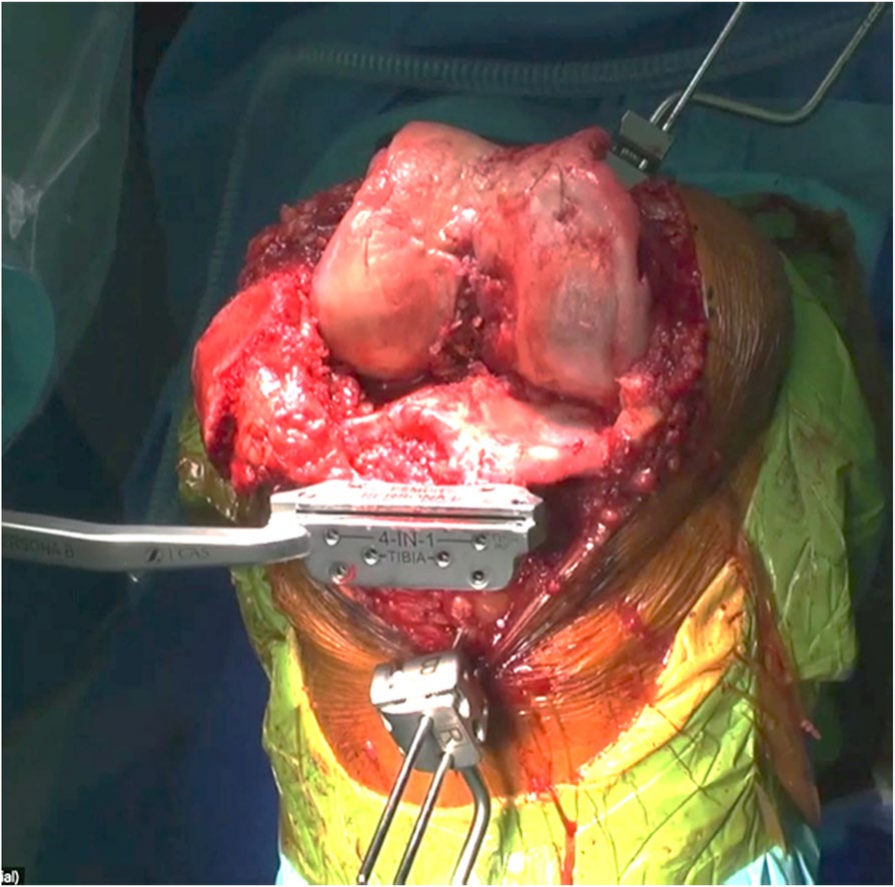
Standard surgical exposure prior to bone cuts

The standard surgical exposure suffices just to expose the anterior aspect of the tibia to ensure juxtaposition of the tibial cutting block of the robot arm. Any further exposure of the anteromedial tibia subsequently was performed as a mean of posteromedial release. Figure 1 shows our standard surgical exposure without any soft tissue release.

All large and accessible osteophytes were removed. It is of note that in this group of patients the standard array trackers were placed within the standard incision. The pre-cut balancing plan was made with the robot after soft tissue laxity was incorporated into the balance algorithm and after assessment in terms of the range of motion with varus and valgus loading.

In all cases, the femoral and tibial bone cuts were aligned perpendicular to the mechanical axis (0 degrees varus valgus). Balancing equation aimed to ensure at least 19 mm of joint space in the medial compartment for a valgus deformity or the lateral compartment for a varus deformity. The opposite compartment was accepted whenever the tighter joint space was observed, as the surgeon was prepared to perform the required soft tissue releases to ensure balance after bone resection and removal of remaining osteophytes.

Once the proximal tibial and distal femoral cuts were made and validated, the remaining osteophytes were removed and the extension space assessed, with the spacer block equivalent to 19 mm space. The aim was to ensure sufficient space in one compartment to accommodate the smallest spacer block and ensure that extension was between 0 and 10 degrees. If the medial compartment was tight, then a posteromedial release would be performed as needed to effect balance and fully accommodate the spacer in both compartments. If the lateral compartment was tight, then a fenestrated posterolateral capsular release plus or minus PCL sacrifice was done as needed to achieve balance and fully accommodate the spacer. These releases would be documented. The FuZion device was then used to ensure a balanced flexion space and was tensioned by using its ratchet system in 95 degrees of flexion. The robot requires the knee to be between 90 and 95 degrees of flexion at this point of tensioning to register the required rotation of the femoral component to balance the flexion space. This rotation of the femoral component was then incorporated into the flexion balance algorithm, and final flexion balance was confirmed, ensuring at least 19 mm medial and lateral joint spaces in flexion. The final femoral 4 in 1 bone cuts were then performed, and the rotation of the femoral component was then confirmed to effect flexion gap balance independent of the transepicondylar or posterior condylar axes.

The trial reduction is the final opportunity to ensure balance is achieved and if further releases are required. This is particularly relevant for sagittal balance and flexion range. If required, the PCL may be fenestrated if any tibial lift off is observed. After the implantation of true prosthesis, the final balance through the range was assessed and documented.

### Data analysis and statistical analysis

We used descriptive statistics to report the incidence of each category of cases treated. All statistical analyses were performed by using Microsoft® Excel (v.16.45).

## Results

**Fig. 2:**
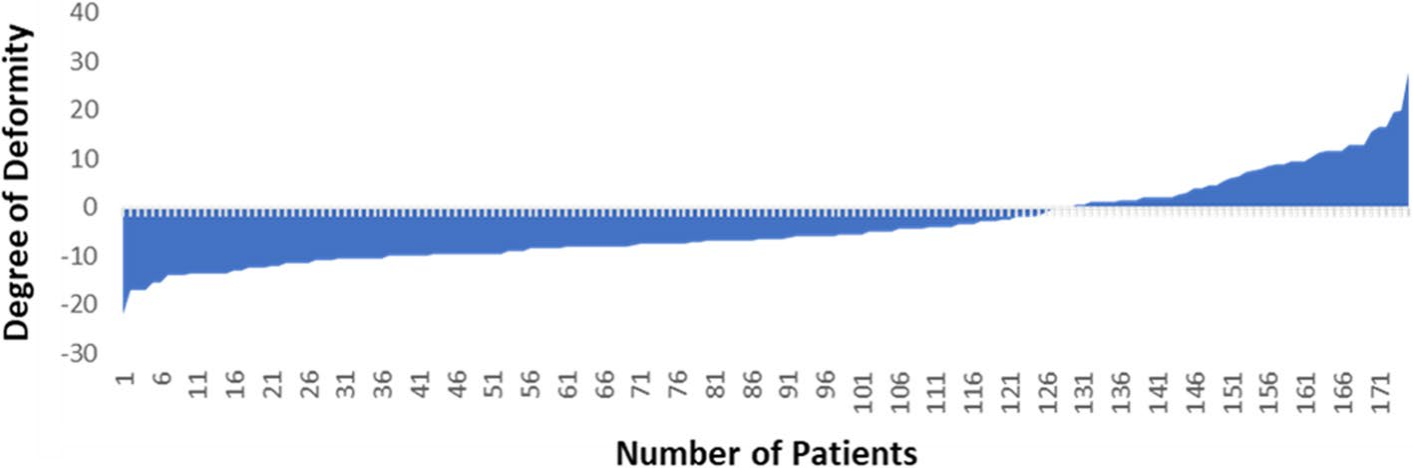
Preoperative HKA deformity

The subjects included 131 female and 44 male patients and were all within the age range from 48 to 89 (average 60 years) years. The soft tissue releases were assessed as a whole group and then in subgroups for varus deformity greater than 2 degrees and valgus deformity more than 2 degrees. The preoperative HKA ranged from 22 degrees varus to 28 degrees valgus, with 71% of the patients presenting with a varus deformity (Fig. 2, Table 1).

**Table 1:** Results of soft tissue releases for varus knee

Releases in Varus HKA < -20 (varus knee)		Total patients- 121
Types of soft tissue releases	*n*	Percentage
Nil releases	86	71%
Fenestrated PCL	24	20%
PCL Sacrificed	7	6%
Medial releases	4	3%

**Fig. 3:**
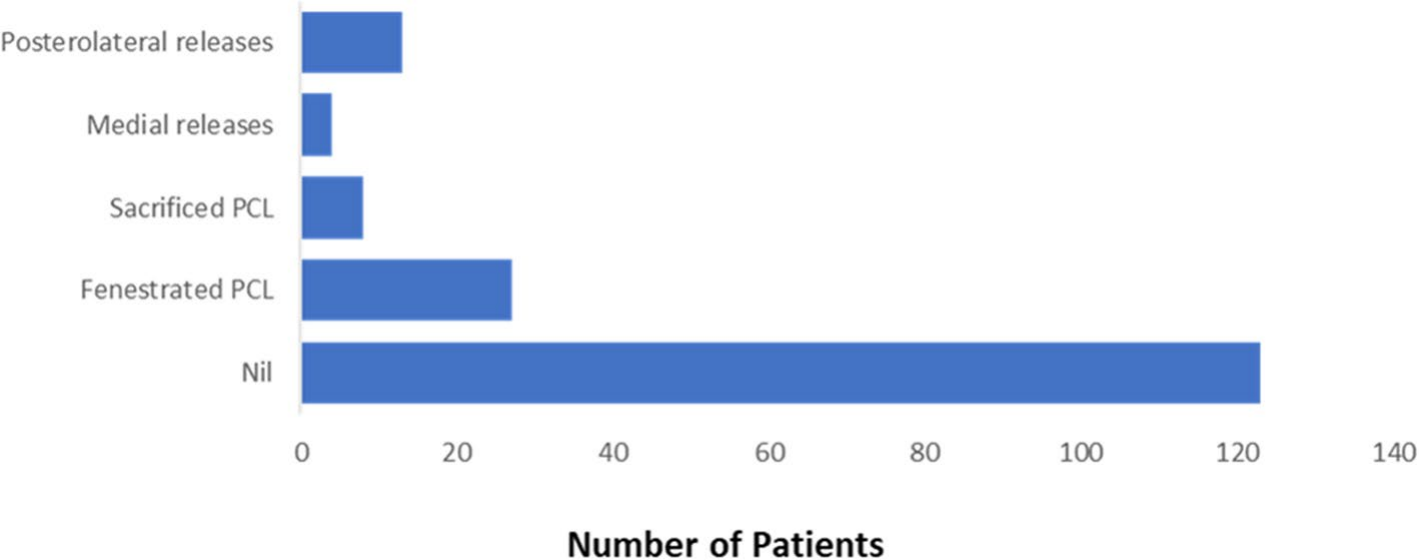
Soft tissue releases performed

For the whole group, no soft tissue release was required in 123 patients (70.3%). In 52 patients (29.7%), some form of soft tissue release was needed to ensure balance. The soft tissue releases performed included small fenestrated releases of PCL in 27 patients (15.4%), sacrifice of PCL in 8 patients (4.5%), posteromedial releases in 4 patients (2.3%) and posterolateral releases in 13 patients (7.4%) (Fig. 3). Included in this patient group requiring soft tissue releases were 4 patients that also needed a bone re-cut (3 femurs, 1 tibia). In the remaining 171 patients, their initial bone resections were validated by ROSA and found to be within the 1.5 mm accuracy zone set by the robot software.

The need for soft tissue releases was higher in patients presenting with valgus knee deformities (Table 2). In patients whose coronal deformity was greater than 2 degrees varus or valgus, the soft tissue release rate was 29% in varus knees) against 53% in valgus knees.

**Table 2:** Result of soft tissue releases for valgus knees

**Releases in valgus HKA > 2° (valgus knee)**		**Total patients (*n* = 32)**
Types of soft tissue releases	***n***	Percentage
Nil releases	15	47%
Fenestrated PCL	3	9%
PCL sacrifice	1	3%
Posterolateral releases	13	41%

The patella was not resurfaced in 137 patients (78%) and a lateral release was required in 18 patients (10%). A lateral release was needed in 6 patients (4%) in those whose patella was not resurfaced. Four of the patients receiving patella resurfacing required augmented tantalum patella due to severe patellofemoral arthritis. The tibial polyethylene inserts were cruciate retaining CR in 131 patients (75%), medial congruent MC in 42 patients (24%), ultra-congruent UC in 1 patient and constrained posterior stabilised CPS in 1 patient. Outcomes to date included no revisions or impending revisions, 2 MUAs (1%), and Oxford knee scores that averaged 40 at 6 months.

## Discussion

Soft tissue releases are utilized *ala carte* and the current use of the technique has the potential to optimize balance and outcomes. A study by Plascos et al. [1] reported that robotic-assisted gap-balancing required less soft tissue release compared to robot-assisted measured resection and conventional measured resection. In the study, 31% out of 615 robotic-assisted gap balancing required one or more soft tissue releases. Similarly, our study reported that 29.7% of the patients that had robotic TKA required a soft tissue release. Clark et al. [4] reported on the significant short-term benefits of robotic TKA as compared with conventional TKA, as indicated by the decreased need for soft tissue releases, in particular, improved range of motion and decreased pain scores on day 1 and a reduction in narcotic use on day 2. Total reduction in morphine-equivalent dosage was also significantly lower, thereby facilitating early mobilization and discharge, with a significant reduction in the length of hospital stay. These early benefits of minimizing soft tissue releases during TKA, however, did not translate to any long-term benefits as there was no difference between groups at 2 years in terms of the Oxford knee score or forgotten knee score.

With the robot-assisted TKA, we could avoid unnecessary soft tissue release optimizing coronal alignment to the mechanical axis to achieve a balanced knee. However, as in study by Morcos et al. [5] and other reports, no difference was identified in patient-reported outcomes after 1 year no matter minimal or extensive soft tissue releases were used in TKA.

**Fig. 4:**
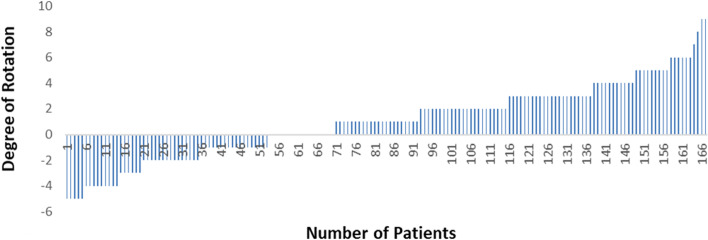
Fenestrated PCL performed using a size 15 blade

In our study, we considered small fenestrated releases of PCL (Fig. 4) and PCL sacrifices for the soft tissue release. The total percentage of patients in these subgroups was 19.9%. Some elected to sacrifice the PCL routinely and substitute with a cam post PS (posterior stabilizer) design or utilize an MC (medial congruent) poly insert and, as a result, the actual rate of soft tissue releases required for correction in the coronal plane was only 9.7%. We believe that preserving the PCL provides a leverage to retain, fenestrate, or remove to achieve better balance of the knee in flexion and extension (Fig. 3).

More patients with valgus knee deformity required soft tissue release in comparison with patients with varus knee deformity (53% vs. 29%). Based on our sub-analysis, we noted that a preoperative HKA greater than 12 degrees was more likely to require a soft tissue release. For patients with a valgus deformity less than 6 degrees the release rate was 0%, for valgus between 6 to 12 degrees the release rate was 22% and for 12 degrees or more the rate of release needed to achieve balance was 67%. This value can serve as a predictor in future to determine the need for soft tissue releases if an MA philosophy is followed. Furthermore, we acknowledge some authors might consider the utilization of a lateral parapatellar approach in more severe valgus deformities but for standardization across our cohort, we used the medial parapatellar approach in all patients regardless of presenting deformity. There was no conversion to higher constraint prosthesis in any of our patients intraoperatively.

**Fig. 5:**
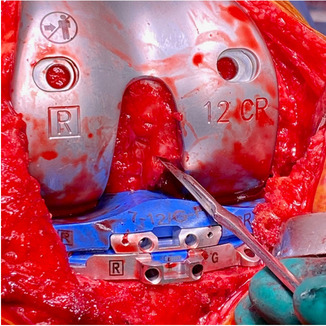
Rotation of femoral component from TEA

Lateral retinacular releases were performed if the patella was not tracking centrally in the trochlear groove at final reduction and 10% of our patients required lateral retinacular release, and 4% were from the non-resurfaced group. The effect of TKA component positioning could contribute to patella mal-tracking [6]. We routinely perform CT as per Perth Protocol after 6 weeks for all our patients to ensure TKA components are in desired positions (Fig. 5 and Table 3). Relative to the trans epicondylar axis, 74% of cases were aligned, 18% were externally rotated more than 3 degrees to the TEA and 8% were internally rotated more than 3 degrees to the TEA.

**Table 3:** Rotation of femoral component from TEA (+ 3° = External rotation, -3° = Internal rotation)

Degrees of rotation	*n*	Percentage
0 ± 3	123	74%
> + 3	30	18%
< -3	14	8%

Firer et al. reported that, in conventional instrumented TKA, no soft tissue releases were secured [7] when ligament balance was secured completely by bone cuts and implant placement to accommodate the soft tissues. PCL was removed in all patients. Bone cuts were made based on a template, depending on the degree of deformity calculated from the pre-operative X-ray by using a gap balancing technique for both flexion and extension. However, no objective ligament balancing assessment was conducted intraoperatively due to the debate on what is the appropriate value of tension [7]. In this study, a balanced knee depended on the ‘surgeons feel factor’ or ‘Surgeon-defined Assessment’ (SDA). MacDessi et al., in their paper, concluded that SDA was a poor measure of soft tissue assessment in TKA [8].

Robot-assisted TKA optimizes the assessment of ligament balance but there are other methods published, such as mechanical tensioners and ‘kinetic sensor’ and second-generation electronic devices. All these devices could reportedly provide objective quantitative assessment of the soft tissue envelope intraoperatively, thus allowing for appropriate releases to ensure the knees are balanced and stable [9–11]. Elmallah et al. compared the experience of a surgeon with 30-years of experience to balance TKAs with sensor-guided TKAs, where releases were allowed to optimize balance with the sensor values. Sensor readings provided feedback to the performance of soft tissue releases and improve balance in TKAs well above the surgeon's feel [12]. A meta-analysis by Batailer et al. reviewed 27 publications with maximum follow-up time of 26 months [13]. Standard surgeon assessment of knee balance was a poor predictor of the true soft tissue balance when compared to sensor data guidance, but prospective comparative data found no demonstrable difference in clinical outcomes, the range of movement or complications at 1 year [13].

Moore et al. reported an average of 3.03 surgical corrections per patient using ‘Verasence’ to achieve balance [14]. This included a total of 331 MCL Pie-crusting, 69 Arcuate releases, 35 posterior capsule releases, 13 ITB releases and 19 popliteus releases [14]. With the robot-assisted TKA, pre-planning and assessment of ligament balance before bone cuts are made can decrease the soft tissue releases required for balance as projected in our study.

With early navigation, Goudie et al. reported that 5 (2.2%) patients out of 224 TKA required releases (4 medial and 1 lateral) using computer navigated TKA [15]. Deformity ranged from 25 degrees of varus to 27 degrees of valgus. Computer-navigated TKA provided accuracy of bone cuts and component alignment [16] but there was no objective assessment of a balanced knee apart from the ‘surgeons feel factor’. MacDessi et al. already concluded that surgeon defined assessment (SDA) is a poor measure of soft tissue assessment in TKA [8].

Robot used in TKA is an advanced navigation machine that provides features of computer-navigated TKA and soft tissue assessment, which is the key factor to achieving a balanced knee.

**Fig. 6:**
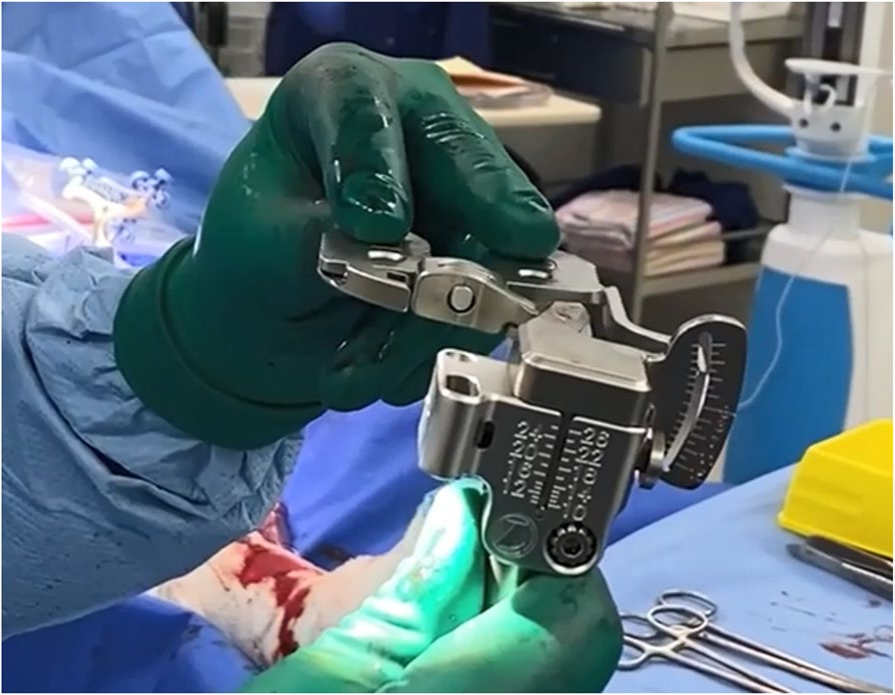
FuZion device for ligament balancing

To date, no agreement has been reached regarding the right amount of force required for joint distraction and, in most cases, it depends on a surgeon’s preference. The knee forces vary from extension to flexion and soft tissue balance changes depending on the joint distraction forces being applied [17]. The strength of joint distraction force is essential in the assessment of soft-tissue balance [17]. Conventional ligament spreaders are being used but this method can cause oversized and asymmetrical gaps [18]. In our series, the FuZion device (Fig. 6) was used to quantitatively measure the opening of medial and lateral space when forces were being applied gradually and to ensure a balanced flexion gap. With this information, appropriately measured bone cuts can be made.

The drawback of this study was that there were no control cohorts for proper comparison. Also, the number of surgeries performed was few. However, the strength of this study was prospectively collected data in a consecutive series, all with a reproducible technique demonstrating and establishing a baseline for required soft tissue release in a mechanically aligned TKA with flexion gap balancing in a robot-assisted TKA.

## Conclusion

Robot-assisted TKA has been available for over 5 years and this important study added to the body of knowledge by investigating the required soft tissue releases using robot-technique in a mechanically aligned flexion gap balanced TKA. Based on bone cut precision, soft tissue parameters delivered by the robot algorithm allows the surgeon to titrate the soft tissue releases needed to achieve balance and in our series a release of any form was required in 29% of cases with more than half of these only involving the PCL.

A prospective randomized control study is required to further evaluate the differences between robot and non-robot input in balancing the soft tissues.

## Data Availability

The data that support the findings of this study are available from Nepean Blue Mountain Local Health District, but restrictions apply to the availability of these data, which were used under license for the current study, and so are not publicly available. Data are however available from the authors upon reasonable request and with permission of Nepean Blue Mountain Local Health District.
